# Using ^1^H-Magnetic Resonance Spectroscopy to Evaluate the Efficacy of Pharmacological Treatments in Parkinson’s Disease: A Systematic Review

**DOI:** 10.3390/ijms26199351

**Published:** 2025-09-25

**Authors:** Lilla Bonanno, Miriana Caporlingua, Jole Castellano, Angelo Quartarone, Rosella Ciurleo

**Affiliations:** IRCCS Centro Neurolesi “Bonino-Pulejo”, Via Palermo S.S. 113, Contrada Casazza, 98124 Messina, Italy; lilla.bonanno@irccsme.it (L.B.); miriana.caporlingua@irccsme.it (M.C.); angelo.quartarone@irccsme.it (A.Q.)

**Keywords:** brain metabolite, cannabinoids, dopamine agonists, levodopa, MAO-B inhibitors, neurochemical biomarkers, neuroprotection, Parkinson’s disease, proton magnetic resonance spectroscopy, therapeutic efficacy

## Abstract

Parkinson’s Disease (PD) is the fastest-growing neurological disorder, characterized by the degeneration of dopaminergic neurons. Treatments remain symptomatic, and objective biomarkers for therapeutic response are lacking. This review aims to evaluate the potential of Proton Magnetic Resonance Spectroscopy (^1^H-MRS) to provide objective and reproducible biomarkers for monitoring treatment response in PD. This systematic review followed PRISMA guidelines. Articles were searched in PubMed, Web of Science, Scopus, and Embase, and studies employing ^1^H-MRS to evaluate pharmacological treatments in PD were included, analyzing pre- and post-treatment changes. Six studies were included, investigating cannabinoids, dopamine agonists, monoamine oxidase B inhibitors, and levodopa. Key metabolites analyzed were N-acetylaspartate, Creatine, Choline, myo-Inositol, and Glx (glutamate+glutamine). Increases in NAA, a marker of neuronal integrity and mitochondrial function, suggested neuroprotective mechanisms of dopaminergic drugs, while stable Cho and mI levels, markers of membrane metabolism and inflammatory processes, suggested limited short-term responsiveness. This is the first systematic review evaluating ^1^H-MRS for monitoring neurometabolic changes induced by pharmacological treatments in PD. Observed metabolite changes appear to reflect treatment mechanisms and potential neuroprotective properties. Findings suggest that ^1^H-MRS may serve as an objective biomarker for assessing therapeutic efficacy and potential neuroprotective drug effects, although further studies are needed to confirm its clinical utility.

## 1. Introduction

Parkinson’s Disease (PD) is a progressive neurodegenerative disorder characterized by the selective loss of dopaminergic neurons in the Substantia Nigra (SN) pars compacta, resulting in a marked depletion of dopamine levels in the putamen of the dorsolateral striatum. This neurochemical imbalance disrupts the modulation of the direct and indirect pathways within the cortico-striato-thalamo-cortical circuits, ultimately resulting in both motor and non-motor symptoms. This dopaminergic deficit disrupts cortico-striato-thalamo-cortical communication and ultimately gives rise to the cardinal motor and non-motor manifestations of the disease [1]. The main motor symptoms include bradykinesia, rigidity, resting tremor, and gait and balance disorders. Non-motor symptoms include autonomic dysfunction, cognitive and neurobehavioral abnormalities, sleep disorders and sensory disturbances such as anosmia, paresthesias, and pain. These symptoms, often present in the early stages, significantly contribute to the impairment of patients’ health-related quality of life [2,3,4].

Despite extensive research, the etiopathogenesis of PD is still not fully understood. According to current knowledge, the key factors contributing to neurodegeneration in PD include oxidative stress, neuroinflammation, dysregulation of cerebral proteostasis, apoptotic dysregulation, and impaired autophagy [5,6]. Oxidative stress induces cellular damage through the excessive production of reactive oxygen species (ROS), which accumulate within cells and damage proteins, lipids, and nucleic acids. In dopaminergic neurons of the SN, ROS progressively accumulate, leading to a gradual loss of neuronal function [7]. In parallel, neuroinflammation, mediated by the activation of glial cells (microglia and astrocytes), contributes to neuronal degeneration through the release of pro-inflammatory cytokines that disrupt and damage the neuronal microenvironment [8]. Dysregulation of cerebral proteostasis, especially synaptic, is also considered to be one of the main causes of neuronal death in PD. This dysregulation may result in abnormal aggregation of proteins, disruption of their utilization processes and hyperactivation of individual proteolytic systems [9]. Among the proteins most strongly implicated, α-synuclein holds a central role in the pathogenesis of the disease. Autophagy inhibition promotes the accumulation of α-synuclein in the cell that can be not only a consequence, but also the cause of reduced autophagy, since insoluble protein aggregates are able to inhibit the activity of many proteolytic enzymes [10]. An additional mechanism involved is the activation of calpains, calcium-dependent proteases capable of cleaving α-synuclein into truncated fragments that can aggregate and enhance its neurotoxicity [11].

Beyond its clinical impact, PD is the fastest-growing neurological disorder worldwide. From 1990 to 2015, the number of affected individuals increased by 118%, amounting to 6.2 million cases. Current estimates suggest that this figure could double by 2040, highlighting an increasing impact on healthcare and social systems [12].

Currently, the PD diagnosis relies primarily on clinical observation, with an overall diagnostic accuracy of less than 90%, especially in the early stages of the disease. This is due to the fact that clinical symptoms typically emerge only after a 60–80% loss of dopaminergic neurons [13]. Such a limitation hampers timely therapeutic intervention and underscores the urgent need for reliable biomarkers capable of enabling early diagnosis, tracking disease progression, and monitoring therapeutic response.

Current pharmacological treatments for PD are solely aimed at alleviating symptoms [14]. Levodopa, the most effective agent for managing motor symptoms, acts as a precursor of dopamine, the neurotransmitter deficient in PD. Once it crosses the Blood-Brain Barrier (BBB), it can be taken up by the remaining dopaminergic neurons and converted into dopamine through presynaptic decarboxylation by the enzyme DOPA decarboxylase. To enhance bioavailability and minimize peripheral side effects, levodopa is typically co-administered with peripheral decarboxylase inhibitors, such as benserazide or carbidopa, which do not cross the BBB but block the peripheral conversion of levodopa to dopamine by inhibiting aromatic L-amino acid decarboxylase [15]. Additional therapeutic options include dopamine agonists, monoamine oxidase B (MAO-B) inhibitors, catechol-O-methyltransferase (COMT) inhibitors, and anticholinergic drugs [16]. Dopamine agonists, such as apomorphine, pramipexole, ropinirole, rotigotine, bromocriptine, pergolide, lisuride, and cabergoline, act directly on dopamine receptors, thereby mimicking the endogenous neurotransmitter. MAO-B inhibitors, including selegiline and rasagiline, selectively and irreversibly inhibit both intracellular and extracellular MAO-B. This reduces or delays the breakdown of dopamine into dihydroxyphenylacetic acid (DOPAC) and hydrogen peroxide. Additionally, they inhibit dopamine reuptake from the synaptic cleft, thereby increasing and prolonging its availability [15]. Safinamide is a highly selective and reversible MAO-B inhibitor. It blocks sodium and calcium channels with consequent inhibition of excessive glutamate release [17]. COMT inhibitors, such as entacapone, tolcapone, opicapone, inhibit the COMT enzyme, reducing the peripheral conversion of levodopa to 3-O-methyldopa. Tolcapone acts both peripherally and centrally [18], whereas entacapone exerts its effect only peripherally [19]. The addition of a COMT inhibitor to levodopa therapy combined with carbidopa or benserazide reduces the peripheral metabolism of levodopa, prolongs its plasma half-life, and increases the amount available in the brain [20].

Although some agents, such as levodopa, have been explored for their potential neuroprotective or disease-modifying properties, the supporting evidence is still inconclusive [21,22].

On the other hand, laboratory studies have provided evidence that rasagiline and selegiline exhibit neuroprotective effects not only due to their antioxidant and levodopa-sparing properties, but also through their anti-apoptotic actions [23,24,25].

Several experimental results confirm that dopamine agonists, such as rotigotine, pramipexole, ropinirole, pergolide, apomorphine and bromocryptine, possess inherent and distinct antioxidant, anti-apoptotic, neurotrophic, and anti-fibril formation properties [15,25,26]. Preclinical and translational studies suggest that MAO-B inhibitors, such as rasagiline and selegiline, may confer neuroprotective benefits. These effects appear to extend beyond their dopaminergic action, encompassing antioxidant activity and the ability to attenuate pro-apoptotic pathways [23,24,25].

Likewise, dopamine agonists (e.g., ropinirole, rotigotine, pergolide, pramipexole, apomorphine) have been reported to influence multiple mechanisms relevant to neurodegeneration, including antioxidant actions, trophic support for neurons, and inhibition of toxic protein aggregation [15,25,26]. It has also been suggested that amantadine may exert a neuroprotective effect through the inhibition of glutamatergic N-methyl-D-aspartate (NMDA) receptors [27].

Novel therapeutic strategies are being developed to target α-synuclein pathology, including monoclonal antibodies, vaccination approaches, anti-aggregation molecules, c-Abl kinase inhibitors (e.g., nilotinib, K0706), and agents promoting protein clearance. Other promising disease-modifying candidates include glucagon-like peptide-1 (GLP-1) receptor agonists [28].

The development of disease-modifying treatments for PD has been significantly constrained by the absence of objective biomarkers capable of accurately evaluating neuroprotective effects [29]. Although ongoing clinical trials primarily utilize cognitive and motor assessments, along with neuroimaging methods like positron emission tomography imaging (PET), these tools have notable limitations and do not provide direct diagnostic insight. Conventional endpoints, such as the Unified Parkinson’s Disease Rating Scale (UPDRS), widely used in clinical trials to evaluate the effectiveness of experimental therapies, are limited by variability, lack of objectivity, and their inability to distinguish symptomatic from disease-modifying actions [30].

However, a promising new opportunity to identify suitable biomarkers for assessing the neuroprotective efficacy of PD drugs is the metabolomics, as it can detect a wide range of metabolite, particularly those related to mitochondrial function and structure, in serum and/or cerebrospinal fluid [31].

**Figure 1:**
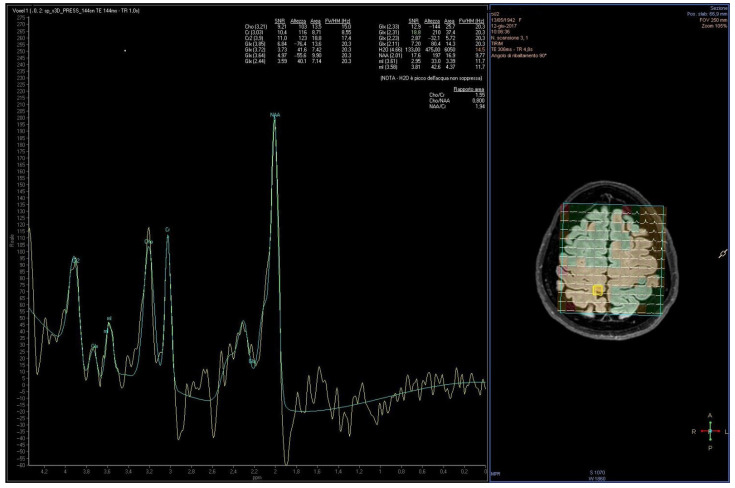
Representative ^1^H-MRS spectrum acquired from the motor cortex of PD patient by Achieva Philips Medical System software. Left: measured spectrum (green line) with labeled metabolite peaks, including N-acetyl aspartate, creatine, choline, myo-inositol, and glutamate/glutamine. The yellow line represents the difference between the measured signal and the model fit. Right: voxel selected for analysis (yellow box) overlaid on the anatomical brain image, with the spectroscopic grid (green) and tissue segmentation maps (colors) used to guide placement. Reprinted with permission from ref. [36]. Copyright 2022 by the authors. Licensee MDPI, Basel, Switzerland. This article is an open access article distributed under the terms and conditions of the Creative Commons Attribution (CC BY) license (https://creativecommons.org/licenses/by/4.0/, accessed on 15 September 2025).

Proton Magnetic Resonance Spectroscopy (^1^H-MRS) is a non-invasive MR technique that does not generate anatomical images (as MRI) but quantifies metabolite signals from a defined brain volume (voxel/VOI), offering a neurochemical profile that may reflect pathological changes associated with neurodegeneration [32]. Each metabolite resonates at a characteristic frequency, and its peak area in the spectrum is proportional to concentration, within technical/biological constraints (sequence, TE/TR, relaxation, coil loading, J-coupling). However, metabolite relationships are complex and influenced by various technical and biological factors, including pulse sequences, relaxation times, coil coupling, and J-modulation effects. To obtain clinically meaningful spectra, it is essential to localize the source of the signals, thereby acquiring data exclusively from a specific brain region. The most commonly used localization techniques include single-voxel spectroscopy, which targets a defined volume of interest (VOI) (typically 2–3 cm^3^), and multi-voxel magnetic resonance spectroscopic imaging (MRSI), which simultaneously acquires spectra from multiple voxels (minimum volume ~0.5–1 cm^3^). Because water is present in the brain at much higher concentrations than any other metabolite, its signal, much more intense, must be suppressed using specific Magnetic Resonance (MR) sequences. Localized ^1^H-MRS with water suppression can be performed using either short or long echo times [33,34,35]. A schematic example of VOI placement and a representative ^1^H-MRS spectrum with annotated peaks is provided in Figure 1.

Among the major metabolites detectable by ^1^H-MRS are: N-acetylaspartate (NAA), a marker of neuronal density and axonal integrity; Choline (Cho), associated with membrane turnover; Myo-inositol (mI), considered a glial marker; Creatine (Cr), involved in cellular energy metabolism; and the glutamate/glutamine complex (Glx), which plays a key role in excitatory neurotransmission [37,38,39].

A growing body of evidence has demonstrated significant alterations in the levels of neurometabolites, including NAA, Cho, Cr, glutamate (Glu), mI and gamma-amminobutyric acid (GABA), in specific brain regions of PD patients compared to healthy control (HC) subjects [40,41,42,43,44,45,46,47,48,49,50,51]. These results suggest that ^1^H-MRS may serve as a dynamic biomarker. Altered metabolite profiles have been correlated with disease duration [52], clinical severity [53], and response to pharmacological treatments [36], indicating the potential utility of this imaging technique in early diagnosis and in monitoring of disease progression and therapeutic outcomes.

This review aims to critically evaluate the application of ^1^H-MRS as an outcome measure for assessing the efficacy of pharmacological treatments in PD. Specifically, it aims to determine whether ^1^H-MRS can provide objective and reproducible biomarkers of treatment response, thereby improving the understanding of dopaminergic drug mechanisms and facilitating the differentiation between symptomatic relief and potential disease-modifying effects.

## 2. Methods

This systematic review was conducted in accordance with the PRISMA (Preferred Reporting Items for Systematic Reviews and Meta-Analyses, 2020) guidelines to ensure transparency and reproducibility in the identification, selection, and evaluation of studies [54].

A comprehensive literature search was performed using four major electronic databases: PubMed, Web of Science, Scopus, and Embase, without temporal restrictions. The search strategy employed a combination of keywords, including Parkinson’s disease, proton magnetic resonance spectroscopy, pharmacological treatment and efficacy, drug therapy, and pharmacotherapy, with the aim of identifying original research articles that utilized ^1^H-MRS to investigate neurometabolic changes induced by pharmacological treatments in patients with PD.

Following duplicate removal, articles not published in English were excluded. Subsequently, two independent reviewers screened the titles and abstracts of the retrieved articles in parallel to assess eligibility and potential risk of bias. Any disagreements that occurred during the screening and data extraction processes were addressed through discussion until they reached an agreement. Studies deemed clearly irrelevant were excluded at this stage. Full-text articles of potentially eligible studies were then assessed for compliance with the predefined eligibility criteria. Studies were excluded if they lacked relevant data, had an inappropriate study design, used imaging techniques other than ^1^H-MRS, or if the article had been withdrawn.

**Figure 2:**
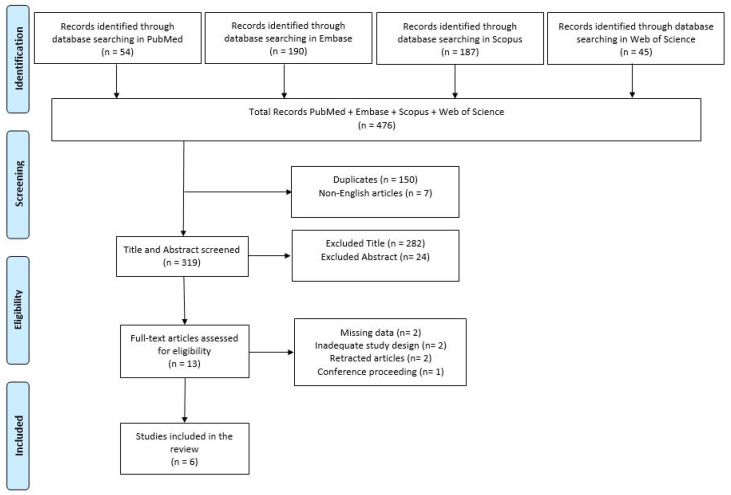
Preferred Reporting Items for Systematic Reviews and Meta-Analyses (PRISMA) diagram. Diagram based on PRISMA 2020 guidelines detailing the selection strategy employed in the current study.

The full study selection process is illustrated in the PRISMA flow diagram (Figure 2).

The research question was structured using the PICO (Population, Intervention, Comparison, Outcome) framework to guide the selection process [55]:

Population (P): patients with a clinical diagnosis of PD;

- Intervention (I): pharmacological treatments, including symptomatic and potentially disease-modifying therapies;
- Comparison (C): HC subjects (i.e., individuals without PD and not undergoing pharmacological treatment), or within-subject comparisons between treated patients and their pre-treatment baseline condition;
- Outcome (O): quantitative changes in cerebral neurometabolic profiles measured by ^1^H-MRS, with a focus on specific metabolites such as NAA, Cho, Cr, mI, Glx, and GABA, considered as potential indicators of treatment response.

*Inclusion Criteria*

Studies were deemed eligible if they fulfilled all of the following criteria:

- Included participants with a clinically diagnosed PD;
- Investigated the effects of pharmacological treatments, either symptomatic or potentially disease-modifying;
- Employed ^1^H-MRS as the imaging modality to measure at least one of the following neurometabolites: NAA, Cho, Cr, Glx, mI, or GABA;
- Included either a HC group and a pre- versus post-treatment comparison within the PD group;
- Published in English.

*Exclusion Criteria*

Studies were excluded if they met one or more of the following criteria:

- Conducted on animal models or cell lines;
- Employed neuroimaging techniques other than ^1^H-MRS (e.g., phosphorus MRS);
- Did not evaluate pharmacological treatment efficacy through ^1^H-MRS;
- Conference proceeding without accompanying full-text articles;
- Lacked original quantitative data on pharmacological treatment effects assessed via ^1^H-MRS;
- Applied ^1^H-MRS for diagnostic or comparative purposes, without assessing therapeutic outcomes;
- Investigated non-pharmacological interventions;
- PD patients already undergoing treatment without baseline (pre-treatment) metabolic assessment;
- Retracted articles;
- Not published in English.

*Risk of Bias Assessment*

The risk of bias in the studies included in this systematic review was assessed using the Quality Assessment Tool for Controlled Intervention Studies, in accordance with the guidelines proposed by the National Institutes of Health (NIH). Two reviewers independently rated each study after a pilot calibration exercise, recording item-level judgments (Yes/No/NR/NA) on a standardized form. Discrepancies were resolved by discussion; when required, a third senior reviewer adjudicated. Final overall ratings (Good/Fair/Poor) were derived by consensus. Item-level results are reported in Table 1.

This tool is based on 14 methodological criteria designed to evaluate critical aspects of study design and conduct. Among these, it evaluates whether the studies were randomized, whether the method of randomization was adequate, and whether treatment allocation was concealed. It also assesses the maintenance of blinding for participants, providers, and outcome assessors, as well as the baseline comparability of the study groups. The tool evaluates both overall and differential drop-out rates, adherence to the intervention protocols and the presence or absence of co-interventions in the groups. Additional criteria include the use of valid and reliable outcome measures, adequacy of the sample size to detect treatment effects, pre-specification of primary analyses and subgroups comparisons, and whether an intention-to-treat analysis was conducted for randomized participants. Overall, the NIH tool enables a structured and transparent evaluation of potential sources of bias, providing insights into the strengths and limitations of the studies reviewed.

## 3. Results and Discussion

### 3.1. Study Selection

A total of 476 records were identified through database searches (PubMed = 54, Scopus = 187, Embase = 190, Web of Science = 45). After removing 150 duplicates and 7 non-English articles, 319 records were screened by title and abstract.

Of these, 282 records were excluded based on title screening due to evident irrelevance to the research question.

Subsequently, 24 records were excluded following abstract screening, based on the following reasons:

- Employed neuroimaging techniques other than ^1^H-MRS (e.g., phosphorus MRS) (n = 1);
- Did not evaluate pharmacological treatment efficacy through ^1^H-MRS (n = 6);
- Applied ^1^H-MRS for diagnostic or comparative purposes only, without assessing therapeutic outcomes (n = 11);
- Included PD patients already undergoing treatment without baseline pre-treatment metabolic evaluation (n = 6).

After title and abstract screening, 13 full-text articles were assessed for eligibility. Of these, 7 studies were excluded based on the following criteria:

- Lacked original quantitative data on pharmacological treatment effects assessed via ^1^H-MRS (n = 2);
- Inadequate study design due to inclusion of non-pharmacological interventions (n = 2);
- Retracted articles (n = 2);
- Conference proceeding without an available full-text article (n = 1).

Ultimately, 6 studies fulfilled all inclusion criteria and were included in the qualitative synthesis.

### 3.2. Risk of Bias Assessment

The included studies were assessed using the NIH 14-item Quality Assessment Tool for controlled intervention studies. The analysis revealed a risk of bias primarily related to the following areas:

- Selection bias, due to the lack of or insufficient reporting on randomization and allocation concealment procedures (Q1, Q2, Q3). Only the studies by Mazuel et al. [13] and Chagas et al. [56] explicitly reported randomization; however, only Mazuel et al. [13] described an adequate method. None of the studies provided a clear description of allocation concealment.
- Performance bias, associated with the absence of blinding of participants, providers (Q4), and outcome assessors (Q5). Only the study by Chagas et al. [56] met both criteria. Blinding of outcome assessors was reported in all studies except those by Clarke et al. [57] and Ciurleo et al. [58].
- Attrition bias was evident in the study by Bonanno et al. [36], which reported a high dropout rate and an unassessable differential dropout.

In addition, the absence of intention-to-treat (ITT) analysis across the included studies represents an another potentially significant source of bias that may influence outcome interpretation.

Each study was classified as having “Good,” “Fair,” or “Poor” methodological quality based on the overall pattern of responses to the 14 items (Table 1). The final classifications were as follows: 2 studies were rated as “Good” [13,56]; 3 studies as “Fair” [57,58,59]; 1 study as “Poor” [36], due to the simultaneous presence of non-randomization, partial absence of blinding, and a high dropout rate. In summary, although the included studies demonstrated strengths such as the use of valid outcome measures and good protocol adherence, several methodological limitations were identified. These issues may affect the reliability and interpretation of the findings and should therefore be carefully considered when evaluating the overall strength of the collected evidence.

### 3.3. Study Characteristics

The publication years of the selected studies ranged from 1997 to 2022. Sample sizes varied from 10 to 80 participants. Of the three longitudinal, open-label studies adopting a within-subject design with pre- and post-treatment comparisons [36,58,59], only one included HC at follow-up [36]. The most frequently analyzed brain regions were the putamen and motor cortex. Three studies used a 3 Tesla high-field scanner [13,36,56]. The main brain metabolites detected across the studies were NAA, Cho, Cr, mI, and Glx. The selected articles investigated the following pharmacological treatments: levodopa (1 study), MAO-B inhibitors including rasagiline and selegiline (1 study), cannabidiol (1 study) and dopamine agonists such as apomorphine, ropinirole and pergolide (3 studies). Table 2 summarizes the key study characteristics, MRS outcomes, and study limitations. Details of statistically significant differences in neurometabolite concentrations in PD patients compared to HCs or after treatment are showed in Supplementary Files (Figures S1–S4).

#### 3.3.1. Levodopa

The randomized, blinded, permuted-block study by Mazuel et al. [13] evaluated the neurochemical profile in the putamen of PD patients undergoing levodopa treatment (drug-on) or after withdrawal of levodopa medication (drug-off) compared with HCs to identify dopaminergic therapy-sensitive biomarkers of PD. The study involved 20 Idiopathic Parkinson’s disease (IPD) patients, 19 of whom received a single dose of levodopa, while 1 was treated with dopamine agonists. Among the participants, 14 were also receiving MAO-B inhibitors, and 9 were treated with COMT inhibitors. A control group of 20 age- and sex-matched HCs was also included. MR spectroscopy was performed twice at 1-week intervals, alternately in “drug-off” condition (following withdrawal of dopaminergic therapy) and in “drug-on” condition (after acute administration of levodopa, 200 mg). Single-voxel ^1^H-MRS was performed using a 3 Tesla scanner, with the VOI placed over the bilateral putamen, in axial, coronal, and sagittal sections. The metabolites analyzed included total NAA (tNAA), total Cr (tCr), mI, Cho, and Glx. In the “drug-off” condition, PD patients exhibited significantly reduced levels of tNAA, tCr, and mI compared to HCs. Following acute levodopa administration (“drug-on”), tNAA and tCr levels significantly increased, reaching values comparable to those observed in healthy subjects (Figure S1). At the same time, clinical symptoms were assessed both during drug-ON and drug-OFF condition. PD patients showed significantly lower UPDRS III scores during the ON condition compared to the OFF condition. No significant correlations were found between individual metabolite levels, UPDRS III scores in either condition, and disease duration. However, a weak correlation emerged between Glx levels and UPDRS III scores in the OFF condition in PD patients. These findings support the hypothesis that levodopa induces not only symptomatic effects but also a reversible modulatory impact on neuronal metabolism within the putamen. In contrast, no significant changes were observed in mI, Cho, or Glx between the two conditions. This suggests that glial activity and glutamatergic neurotransmission are not acutely modulated by dopaminergic stimulation, or that such effects may not be detectable by ^1^H-MRS over a short timeframe.

#### 3.3.2. MAO-B Inhibitors

The longitudinal study by Bonanno et al. [36] evaluated the long-term effects of MAO-B inhibitors, specifically rasagiline (1 mg/day) and selegiline (10 mg/day) on neurometabolic profiles in the motor cortex of de novo PD patients using ^1^H-MRS. The study design included a 12-month follow-up period, during which 40 de novo PD patients were enrolled and divided into two treatment groups (20 receiving rasagiline and 20 selegiline), and compared to 40 age- and sex-matched HCs. Spectroscopic scans were acquired using a 3 Tesla scanner with a multi-voxel ^1^H-MRS technique. The VOI was centered bilaterally over the motor cortex.

The metabolites analyzed were NAA/Cr and Cho/Cr. At baseline, PD patients showed significantly reduced NAA/Cr values compared to HCs, confirming early impairment of neuronal function even in the initial stages of the disease. After 12 months of treatment with MAO-B inhibitors, NAA/Cr levels increased significantly, reaching values comparable to those of healthy subjects (Figure S2). Patients were clinically evaluated at baseline and after 12 months of treatment using the Hoehn and Yahr (H&Y) and UPDRS-III scales to monitor disease progression and therapeutic response. Following treatment with rasagiline or selegiline, a significant inverse correlation was observed between UPDRS-III scores and NAA concentrations, which was associated with an improvement in motor performance, while no significant correlation was found with Cho levels in the motor cortex. Although changes in UPDRS-III scores cannot be directly attributed to a neuroprotective effect, their correlation with changes in NAA levels suggests that this metabolic parameter may represent a reliable indicator of therapeutic efficacy and potentially of disease progression slowing. This increase in NAA suggests a potential neuroprotective effect or neuronal metabolic stabilization induced by dopaminergic modulation. In contrast, the Cho/Cr ratio did not show significant changes during the follow-up (Figure S2). Although there was a trend toward reduced Cho levels at baseline, the absence of modifications after treatment suggests that MAO-B inhibitors may not exert a measurable impact on membrane metabolism or glial/inflammatory processes in the short-to-medium term, at least at the cortical level. NAA is thus confirmed as a sensitive biomarker of neuronal integrity, capable of reflecting therapeutic response, while Cho appears less responsive in this specific context.

#### 3.3.3. Cannabidiol

The randomized, double-blind, placebo-controlled trial by Chagas et al. [56] included 21 patients with PD without dementia or psychiatric comorbidities. Participants were divided into three groups of seven subjects each, receiving treatment for six weeks with either placebo, cannabidiol (CBD) 75 mg/day, or CBD 300 mg/day. Assessments were conducted at baseline and follow-up using ^1^H-MRS. The study did not include a HC group.

The study employed single-voxel ^1^H-MRS using a 3 Tesla scanner, with the VOI placed in the bilateral basal ganglia (putamen). The primary aim was to explore the potential neuroprotective effects of CBD by examining the NAA/Cr ratio.

Participants were assessed in respect to motor and general symptoms score (UPDRS); well-being and quality of life (The Parkinson’s Disease Questionnaire, PDQ-39); and possible neuroprotective effects (BDNF and ^1^H-MRS). No statistically significant differences were found in UPDRS scores, plasma BDNF levels or ^1^H-MRS measures (NAA/Cr ratios) between the placebo and CBD-treated groups (both 75 mg/day and 300 mg/day), at baseline or after 6 weeks of treatment. However, the group treated with CBD 300 mg/day showed a significant improvement in quality of life, as indicated by PDQ-39 total scores significantly different from the placebo group. These results suggest a possible effect of CBD in improving measures related to the quality of life of PD patients without psychiatric comorbidities. Although the endocannabinoid system represents a promising therapeutic target, particularly for addressing non-motor symptoms of PD and for its potential neuroprotective effects [60], current metabolic data do not reveal significant changes in markers of neuronal integrity following CBD treatment.

#### 3.3.4. Dopamine Agonists

The short-term pre-post study by Clarke et al. [57] investigated the lentiform nucleus in five patients with moderately severe IPD and five age-matched HC. The aim was to explore neuronal loss in IPD through quantitative assessment of NAA, Cr, and Cho in the lentiform nucleus, and to evaluate changes in Glx levels within the basal ganglia in response to a fast-acting dopamine agonist. Single-voxel ^1^H-MRS was performed using a 1.5 Tesla scanner, with the VOI placed in the putamen and globus pallidus (lentiform nucleus) in the hemisphere contralateral to the most severely affected side, before and 10 min after subcutaneous administration of apomorphine. The analyzed metabolic ratios included Cho, Cr, NAA (absolute quantification) and Glx/Cr. Repeat MRS scans revealed no significant differences in striatal metabolite concentrations (NAA, Cho, Cr) or Glx/Cr ratios between IPD patients and controls, nor before and after apomorphine administration, indicating no MRS-detectable metabolic changes in the basal ganglia in moderate IPD. This study lacks an analysis of correlations between cerebral metabolite concentrations measured by ^1^H-MRS and clinical scale scores used to assess PD severity, an analysis that could have helped clarify the potential value of neurometabolic biomarkers in reflecting patients’ clinical status. However, this omission appears justified by the very short timeframe of the study (a follow-up of only ten minutes), which would not have allowed the detection of clinically significant changes.

The longitudinal study by Ciurleo et al. [58] investigated the effects of ropinirole treatment on the neurometabolic profile of the motor cortex in drug-naïve de novo PD patients using ^1^H-MRS. The study included 20 PD patients naïve to pharmacological treatment and 15 age-matched HCs, evaluated at baseline and after ten months of ropinirole therapy titrated up to 6 mg/day. MRS scans were conducted using a 1.5 Tesla multi-voxel scanner, with the VOI positioned over the corpus callosum to primarily include the white matter and mesial motor cortex of both hemispheres. The metabolic ratios analyzed included NAA/Cr, NAA/Cho, and Cho/Cr. At baseline, PD patients showed significantly lower NAA/Cr and NAA/Cho ratios compared to HCs, along with an increased Cho/Cr ratio. After ten months of treatment, a significant increase in both NAA/Cr and NAA/Cho was observed (Figure S3). Additionally, a highly significant correlation was found between these neurometabolic ratios and the motor sub-scores of the UPDRS, supporting the therapeutic efficacy of ropinirole in improving motor performance. The rise in NAA was interpreted as a partial recovery of neuronal activity or cortical metabolic function, consistent with a neurofunctional effect of the drug. An inverse correlation between NAA levels and clinical severity further supports the hypothesis that ^1^H-MRS biomarkers are sensitive to dopaminergic treatment response. In contrast, Cho/Cr did not show significant changes after treatment (Figure S3), suggesting that the initial Cho elevation may reflect a persistent inflammatory process not modifiable by dopaminergic stimulation alone.

The longitudinal study by Lucetti et al. [59] included 11 de novo PD patients and 11 age-matched HC, aiming to evaluate neurochemical and metabolic changes in the motor cortex before and after six months of treatment with the dopamine agonist pergolide (1 mg three times daily). Single-voxel ^1^H-MRS was performed using a 1.5 Tesla scanner, with the volume VOI placed on the medial surface of the motor cortex, including both the left and right cortical sides. Metabolic ratios investigated included NAA/Cr, Cho/Cr, and mI/Cr. At baseline, PD patients showed lower Cho/Cr and NAA/Cr values compared to HC (Figure S4). No significant relationships were found between Cho/Cr and NAA/Cr ratios and age, sex, motor disability, or disease duration. After six months of pergolide treatment, a significant increase in Cho/Cr was observed, along with a non-significant upward trend in NAA/Cr, suggesting partial metabolic normalization. The absence of significant changes in mI/Cr ruled out a relative decrease in Cr as the cause for the observed increases, indicating a true rise in Cho and NAA levels (Figure S4). The authors suggested that the restoration of Cho/Cr could reflect normalization of membrane turnover, while the increase in NAA/Cr could indicate improved neuronal functionality. PD patients were clinically evaluated one, three, and six months after the beginning of pergolide treatment by UPDRS. After six months of pergolide therapy, significant improvement in UPDRS sub-item II and III was found, indicating enhanced motor performance. In summary, this study suggests that cortical ^1^H-MRS can be a valuable tool for monitoring the impact of dopaminergic therapy on neuronal function.

### 3.4. Methodological Limitations of the Included Studies

Although the selected studies contribute to outlining the potential role of ^1^H-MRS in monitoring the effects of pharmacological treatments in PD, they collectively present several recurring methodological limitations that restrict the generalizability and strength of their conclusions.

A primary limitation concerns the small sample sizes analyzed. Several studies, [13,56,57,58,59] enrolled very few patients, resulting in low statistical power.

A second critical issue relates to the duration of follow-up. Some studies relied on short-term assessments [56,57], which may be insufficient both to capture detectable neurochemical changes and to highlight clinically significant effects, as observed in the study by Clarke et al. [57], who did not analyze correlations between metabolic changes and clinical severity scores due to the very short follow-up period that hindered detection of such effects. On the other hand, in longer-term studies [58,59], the natural progression of the disease may have influenced the spectroscopic data, making it difficult to attribute the observed changes exclusively to the pharmacological intervention. Indeed, in these studies, ^1^H-MRS was not performed at follow-up on HCs.

From a technical standpoint, one of the most frequently encountered limitations involves the localization of VOI. For example, in Lucetti et al. [59] the VOI was positioned over the medial surface of the motor cortex. Given its size, signals from supplementary motor areas may have been included in the analysis. Furthermore, the spectroscopic data did not allow for lateralization between the right and left hemispheres, limiting the interpretability of the results. Another methodological concern was the lack of quantification and correction for cerebrospinal fluid (CSF) content within the VOI, which may have contaminated the MRS signal. It cannot be ruled out that pergolide treatment induced CSF changes that influenced the spectroscopic findings.

Regarding the brain regions investigated, the studies focused exclusively on the motor cortex [36,58,59] and striatum [13,56,57]. These areas are well known for their involvement in motor function and are therefore logical targets for investigating drug-induced neurochemical changes. However, it is noteworthy that these studies did not include the substantia nigra, despite its critical role in the pathophysiology of PD as the primary site of dopaminergic neuron loss. As a result, the changes observed, primarily in the motor cortex, may reflect downstream or compensatory effects rather than direct effects on the key site of neurodegeneration. Conversely, no significant changes in metabolite levels were detected in the striatum following treatment, except in the study by Mazuel et al. [13], possibly due to technical challenges associated with ^1^H-MRS in this region, such as its small size, anatomical complexity, and susceptibility to magnetic field inhomogeneities. Nonetheless, given that PD symptoms primarily arise from substantia nigra dysfunction, the absence of direct measurements in this region limits the ability to fully evaluate a drug’s efficacy in protecting or restoring function at the core pathological site. Additionally, in three studies [57,58,59], 1.5 Tesla scanners were used, which offer lower spectral resolution compared to more advanced 3T systems. Another limitation is the variability among studies in acquisition protocols and post-acquisition analysis (post-processing). Differences in magnetic field strength, voxel placement, spectral parameters, and data processing methods can affect comparability and interpretation of results.

Finally, therapeutic and clinical variability represents another significant limitation. In the study by Mazuel et al. [13] the presence of concomitant medications (e.g., MAO-B and COMT inhibitors) constituted a potential confounding factor that was not adequately controlled. Additionally, this study investigated only the acute effects of levodopa administration, which may not reflect the neurometabolic changes associated with chronic treatment, thereby limiting the clinical relevance of the findings. In other studies, the absence of placebo groups or untreated patients made it difficult to isolate the specific effect of the pharmacological intervention on the neurometabolic changes observed.

### 3.5. Results Interpretation

Numerous studies employing ^1^H-MRS have identified characteristic neurochemical alterations in patients with PD compared to HC. Among the most consistently reported findings is a reduction in NAA ratios, particularly NAA/Cr and NAA/Cho, reflecting impaired neuronal integrity and mitochondrial dysfunction, both of which are central to the pathophysiology of PD. Other metabolites, such as Cho and Glx have shown more variable or nonsignificant alterations across studies, suggesting either a delayed or more heterogeneous involvement in disease progression [61,62,63]. The studies reviewed in this work corroborate these findings, reporting decreased NAA/Cr and NAA/Cho ratios in PD patients at baseline, prior to pharmacological treatment. While the symptomatic efficacy of dopaminergic therapies is well-established, growing interest lies in understanding their potential neurochemical and neuroprotective effects. Treatments such as levodopa, dopamine agonists (e.g., pergolide, ropinirole), and MAO-B inhibitors (e.g., rasagiline, selegiline) have been explored in this context. Increases in NAA/Cr ratios following treatment suggest a potential restoration of mitochondrial function and neuronal integrity, aligning with the proposed mechanisms of action of these agents, including dopaminergic modulation, attenuation of oxidative stress, protection against apoptotic damage, and support for cell survival. NAA, synthesized in neuronal mitochondria via the acetylation of L-aspartate by L-aspartate N-acetyltransferase using acetyl-CoA, serves as a direct marker of mitochondrial activity. Its increase post-treatment may thus reflect mitochondrial protection, counteracting the pro-oxidant effects of reactive species [64]. Among other metabolites, Glx, a marker of excitatory neurotransmission, has not shown treatment-related changes, and similarly, levels of Cho and mI, associated with membrane turnover, gliosis, and neuroinflammatory processes, showed limited or no changes, suggesting that these pathological aspects may not be immediately influenced by dopaminergic therapy or may require longer periods to become apparent. For instance, in the study by Lucetti et al. [59] the reduction in the Cho signal, originating from choline-containing compounds involved in membrane synthesis and from the polar heads of myelin lipids, could be interpreted as a marker of astrogliosis, but may also reflect changes in the chemical composition and/or functional properties of cellular membranes [65]. Consequently, the subsequent increase in Cho signal amplitude following pergolide treatment may indicate a normalization of cellular metabolism and membrane structure, particularly at the lipid-water interface [66].

## 4. Conclusions

Overall, these observations suggest the value of ^1^H-MRS as a non-invasive tool for monitoring brain metabolic changes in response to treatment. The measured metabolites could serve as objective biomarkers to assess therapeutic efficacy and the neuroprotective potential of pharmacological interventions. Spectroscopic markers, particularly NAA, are suggested to be objective indicators of therapeutic benefit and potential neuroprotection. Furthermore, these findings support the hypothesis that PD is a multisystem and neurometabolically heterogeneous disorder, in which distinct neurochemical alterations may coexist and contribute to individual variability in treatment response.

Nevertheless, these results should be interpreted with caution. This review included only six studies, which were characterized by heterogeneous designs, small sample sizes, and notable methodological and technical limitations. The overall quality of the evidence is also influenced by risk of bias, variability in acquisition protocols and selection of brain regions, as well as the predominant use of relative rather than absolute metabolite quantification. To better define the role of ^1^H-MRS in assessing treatment efficacy and possible neuroprotective effects, future studies should involve larger patient samples, longer follow-up, and harmonized methodologies. It would also be beneficial to explore a broader range of brain regions involved in the disease’s pathophysiology to fully capture the neurochemical complexity of PD. For example, ^1^H-MRS measurements in the substantia nigra could provide more direct insights into the neurochemical impact of treatments on the core affected area in PD. Integrating ^1^H-MRS with multidimensional approaches, such as metabolomics, proteomics, and other “omics” technologies, could offer a more comprehensive and systemic perspective on biochemical alterations and treatment responses. Future developments should also include absolute quantification of metabolites to enhance comparability across studies and improve sensitivity in detecting metabolic changes. Only through a methodologically rigorous and multidisciplinary approach can ^1^H-MRS be established as a clinically relevant tool for assessing therapeutic efficacy and neuroprotection in PD.

## Figures and Tables

**Table 1:** Risk of bias assessment using the National Institutes of Health (NIH) Quality Assessment Tool for Controlled Intervention Studies.

Study	Q1	Q2	Q3	Q4	Q5	Q6	Q7	Q8	Q9	Q10	Q11	Q12	Q13	Q14	Quality Rating
**Mazuel et al. [13]**	Yes	Yes	NR	No	Yes	Yes	Yes	Yes	Yes	Yes	Yes	No	Yes	NR	Good
**Bonanno et al. [36]**	No	NA	NA	No	Yes	Yes	No	NR	Yes	Yes	Yes	No	Yes	NA	Poor
**Chagas et al. [56]**	Yes	NR	NR	Yes	Yes	Yes	Yes	Yes	Yes	Yes	Yes	No	Yes	NR	Good
**Clarke et al. [57]**	No	NA	NA	No	NR	Yes	Yes	Yes	Yes	Yes	Yes	No	Yes	NA	Fair
**Ciurleo et al. [58]**	No	NA	NA	No	NR	Yes	Yes	Yes	Yes	Yes	Yes	No	Yes	NA	Fair
**Lucetti et al. [59]**	No	NA	NA	No	Yes	Yes	Yes	Yes	Yes	Yes	Yes	No	Yes	NA	Fair

*NR*, *Not Reported*; *NA*, *Not Applicable.* Criteria: 1. Was the study described as randomized, a randomized trial, a randomized clinical trial, or an RCT? 2. Was the method of randomization adequate (i.e., use of randomly generated assignment)? 3. Was the treatment allocation concealed (so that assignments could not be predicted)? 4. Were study participants and providers blinded to treatment group assignment? 5. Were the people assessing the outcomes blinded to the participants’ group assignments? 6. Were the groups similar at baseline on important characteristics that could affect outcomes (e.g., demographics, risk factors, co-morbid conditions)? 7. Was the overall drop-out rate from the study at endpoint 20% or lower than the number allocated to treatment? 8. Was the differential drop-out rate (between treatment groups) at endpoint 15 percentage points or lower? 9. Was there high adherence to the intervention protocols for each treatment group? 10. Were other interventions avoided or similar in the groups (e.g., similar background treatments)? 11. Were outcomes assessed using valid and reliable measures, implemented consistently across all study participants? 12. Did the authors report that the sample size was sufficiently large to be able to detect a difference in the main outcome between groups with at least 80% power? 13. Were outcomes reported or subgroups analyzed prespecified (i.e., identified before analyses were conducted)? 14. Were all randomized participants analyzed in the group to which they were originally assigned, i.e., did they use an intention-to-treat analysis?

**Table 2:** Summary of included studies evaluating pharmacological treatments in Parkinson’s Disease (PD) using Proton Magnetic Resonance Spectroscopy (^1^H-MRS).

Study	Population	Study Design	Study Aim	Drug	Metabolite (s)	Follow-Up Period (Baseline to Follow-Up MRS)	MRS Technique and VOI	Main Findings	Limits
Mazuel et al. [13]	20 IPD patients; 20 age- and sex-matched HCs.	Randomized, blinded, permuted-block study.	To assess the neurochemical profile in the putamen of PD patients undergoing levodopa treatment (drug-on) or after withdrawal of levodopa medication (drug-off) compared with HCs to identify dopaminergic therapy–sensitive biomarkers of PD.	Levodopa (200 mg acute dose, Modopar).	tNAA tCr mI Glx	Twice at 1 week intervals, alternately in drug-off condition and in drug-on condition.	Single voxel, 3T scanner. Right and left putamen in axial, coronal and sagittal sections.	PD patients in drug-off condition showed significantly lower tNAA, tCr, and mI levels vs. HCs. After levodopa administration, tNAA and tCr increased to near-normal levels. No significant changes were observed in mI, Cho, or Glx between the two conditions.	Acute effect of levodopa, not representative of chronic therapy. Concomitant therapies (MAO-B, COMT inhibitors) potentially confounding.
Bonanno et al. [36]	40 de novo PD patients (20 rasagiline, 20 selegiline); 40 age- and sex-matched HCs.	Longitudinal prospective controlled study.	To evaluate the effects of rasagiline and selegiline on neurometabolic profiles in the motor cortex of de novo PD patients.	MAO-B inhibitors: rasagiline (1 mg/day) and selegiline (10 mg/day).	NAA/Cr Cho/Cr	12 months	Multi voxel, 3T scanner. Motor cortex including both hemispheres.	At baseline, PD patients had significantly lower NAA/Cr ratios than HCs. After 12 months of treatment with either rasagiline or selegiline, NAA/Cr significantly increased to values comparable with HCs. No significant changes were observed for Cho/Cr.	Biological fluctuations of neurometabolite concentrations over time, partly because of aging.
Chagas et al. [56]	21 PD patients (3 groups of 7 subjects: placebo, CBD 75 mg, CBD 300 mg).	Randomized, double-blind, placebo-controlled exploratory trial.	To explore the effects of CBD on motor symptoms, quality of life, BDNF levels, and neurometabolic markers to evaluate potential neuroprotective effects (^1^H-MRS) in patients with PD.	Cannabidiol (CBD) 75 mg/day and CBD 300 mg/day.	NAA/Cr	6 weeks	Single-voxel, 3T scanner. Bilateral basal ganglia (putamen).	No statistically significant changes in NAA/Cr ratios between the CBD and placebo groups at baseline and after 6 weeks.	Small sample size and short follow-up period (6 weeks).
Clarke et al. [57]	5 patients with moderately severe IPD; 5 age-matched HCs.	Short-term pre-post controlled study.	To investigate neuronal loss in IPD by quantifying NAA, Cr, and Cho in the lentiform nucleus, and to assess changes in Glx levels inside the basal ganglia before and after acute dopaminergic stimulation.	Apomorphine (rapidly acting dopamine agonist), single subcutaneous dose.	Cho, Cr, NAA (absolute quantification) Glx/Cr	10–15 min	Single-voxel, 1.5T scanner. Putamen and globus pallidus (lentiform nucleus) in the hemisphere contralateral to the most severely affected side.	No significant differences in striatal metabolite concentrations (NAA, Cho, Cr) or Glx/Cr ratio were observed between IPD patients and controls, nor before and after apomorphine administration.	Very small sample size. Post-treatment assessment performed shortly after drug administration (10–15 min), potentially inadequate to detect metabolic changes.
Ciurleo et al. [58]	20 de novo drug-naïve PD patients; 15 age-matched HCs.	Longitudinal prospective controlled study.	To evaluate neurometabolic changes in the motor cortex of de novo PD patients before and after ropinirole treatment.	Ropinirole (dopamine agonist), 6 mg/day.	NAA/Cr NAA/Cho Cho/Cr	10 months	Multi-voxel, 1.5T scanner. Corpus callous including white matter and mesial motor cortex of both hemispheres.	At baseline, PD patients showed decreased NAA/Cr and NAA/Cho, and increased Cho/Cr ratios vs. HCs. After 10 months of ropinirole therapy, NAA/Cr and NAA/Cho significantly increased.	Small sample size. Possible influence of disease progression on MRSI data at 10-month follow-up. Lack spectroscopic data on HCs at follow-up.
Lucetti et al. [59]	11 de novo PD patients; 11 age-matched HCs.	Longitudinal prospective controlled study.	To investigate neurochemical and metabolic changes in the motor cortex of de novo PD patients before and after 6 months treatment with the dopamine agonist pergolide.	Pergolide (dopamine agonist), 1 mg three times daily.	Cho/Cr NAA/Cr mI/Cr	6 months	Single-voxel, 1.5T scanner. Medial surface of the motor cortex including left and right cortical sides.	At baseline, PD patients showed reduced Cho/Cr and NAA/Cr ratios compared to controls. After 6 months of pergolide therapy, Cho/Cr significantly increased, indicating metabolic normalization; NAA/Cr also increased, though not significantly	VOI centered on the medial surface of the motor cortex, but possible inclusion of supplementary areas. No hemispheric separation in data analysis. Lack of CSF content measurement and correction, with potential MRS signal contamination. CSF alterations induced by pergolide treatment cannot be ruled out. Lack spectroscopy data on HCs at follow-up.

Legend: BDNF: brain-derived neurotrophic factor, CBD: cannabidiol, Cho: Choline, COMT: Catechol-O-Methyltransferase, Cr: Creatine, CSF: Cerebrospinal fluid, Glx: glutamate + glutamine, HCs: Health Controls, ^1^H-MRS: proton magnetic resonance spectroscopy, IPD: idiopathic Parkinson’s disease, MAO-B: Monoamine Oxidase B, MRS: magnetic resonance spectroscopy, MRSI: magnetic resonance spectroscopy imaging, mI: myoinositol, NAA: N-acetylaspartate, PD: Parkinson’s Disease, T: Tesla, tCr: total creatine, tNAA: total N-acetylaspartate, VOI: volume of interest.

## Data Availability

Not applicable.

## Supplementary Materials

The following supporting information can be downloaded at https://www.mdpi.com/article/10.3390/ijms26199351/s1.

## Abbreviations

The following abbreviations are used in this manuscript:

BBB	Blood–Brain Barrier
BDNF	Brain-Derived Neurotrophic Factor
CBD	Cannabidiol
Cho	Choline
COMT	Catechol-O-Methyltransferase
Cr	Creatine
CSF	Cerebrospinal fluid
DOPAC	Dihydroxyphenylacetic Acid
GABA	Gamma-Amminobutyric Acid
GLP-1	Glucagon-like Peptide-1
Glu	Glutamate
Glx	Glutamate + Glutamine
^1^H-MRS	Proton Magnetic Resonance Spectroscopy
H&Y	Hoehn and Yahr
HC(s)	Health Control(s)
IPD	Idiopathic Parkinson’s disease
ITT	Intention-to-treat
MAO-B	Monoamine Oxidase B
MR	Magnetic Resonance
MRI	Magnetic Resonance Imaging
MRS	Magnetic Resonance Spectroscopic
MRSI	Magnetic Resonance Spectroscopic Imaging
NA	Not applicable
NAA	N-acetylaspartate
NIH	National Institutes of Health
NMDA	N-methyl-D-aspartate
NR	Not Reported
PD	Parkinson’s Disease
PDQ-39	The Parkinson’s Disease Questionnaire
PET	Positron emission tomography imaging
PICO	Population, Intervention, Comparison, Outcome
PRISMA	Preferred Reporting Items for Systematic Reviews and Meta-Analyses
ROS	Reactive oxygen species
SN	Substantia Nigra
T	Tesla
tCr	total Creatine
tNAA	total N-acetylaspartate
UPDRS	Unified Parkinson’s Disease Rating Scale
VOI	Volume of interest
