# Supplementary material for: Using 1H-Magnetic Resonance Spectroscopy to Evaluate the Efficacy of Pharmacological Treatments in Parkinson’s Disease: A Systematic Review
Source: Int J Mol Sci. 2025 Sep 25;26(19):9351. doi: 10.3390/ijms26199351 (PMC12525049; doi:10.3390/ijms26199351)
Supplement: Supplementary file 1 [file ijms-26-09351-s001.zip › ijms-3869596-supplementary.pdf]

Supplementary Files

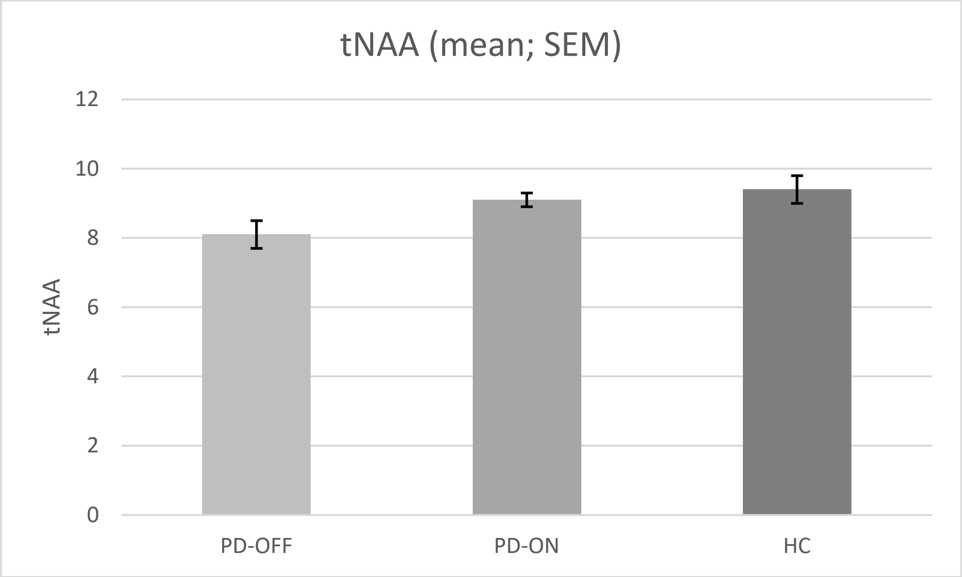

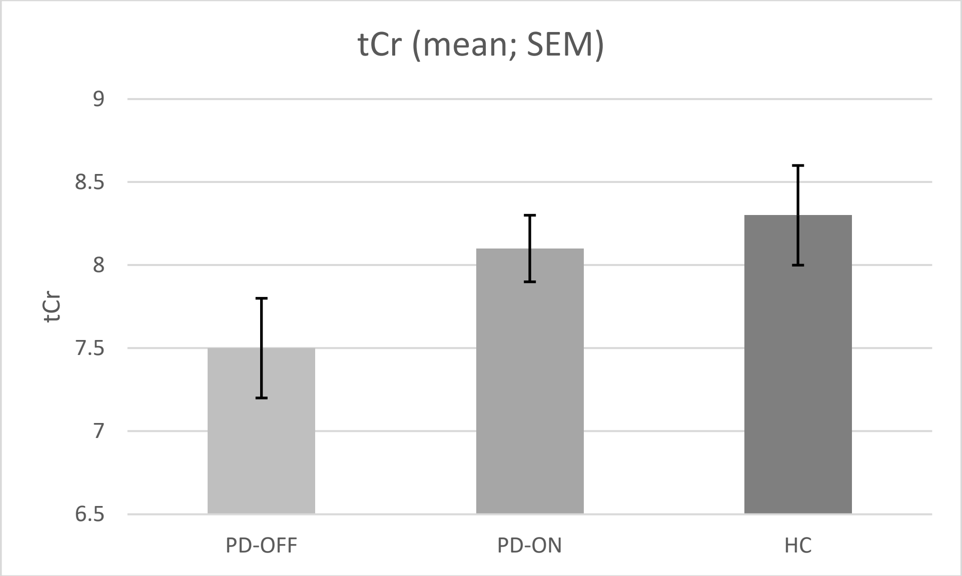

Figure S1. Putamen neurometabolites with acute levodopa (Mazuel et al., Radiology 2016). Bar plots show total N-acetylaspartate (tNAA) (top) and total creatine (tCr) (bottom) measured by single-voxel ^{1}H-MRS at 3 T in the putamen. Data are re-plotted from Mazuel et al.: PD patients (n=20) were scanned OFF medication and again ON after an acute 200 mg levodopa dose; healthy controls (HC, n=20) were scanned once. Bars = mean; error bars = SEM. In PD, both tNAA and tCr were reduced OFF vs HC and increased ON vs OFF, approaching HC levels (significant in the original report, $p < 0.01$).
Legend: PD= Parkinson's disease; HC= healthy controls; OFF/ON, off/on dopaminergic medication.

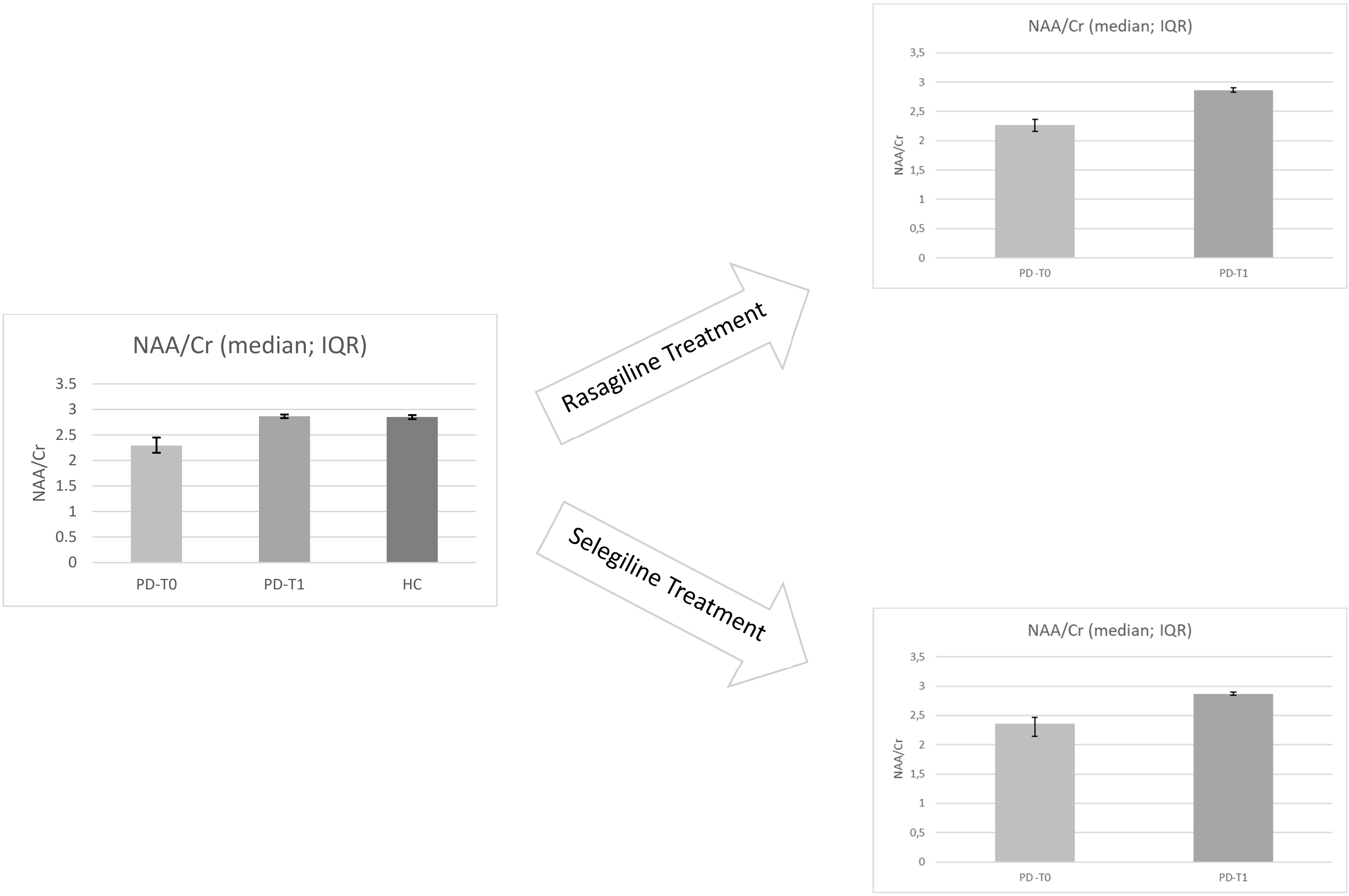
NAA/Cr (median; IQR)
NAA/Cr
3.5
3
2.5
2
1.5
1
0.5
0
PD-T0
PD-T1
HC
Rasagiline Treatment
Selegiline Treatment
NAA/Cr (median; IQR)
NAA/Cr
3,5
3
2,5
2
1,5
1
0,5
0
PD -T0
PD-T1
NAA/Cr (median; IQR)
NAA/Cr
3,5
3
2,5
2
1,5
1
0,5
0
PD -T0
PD-T1

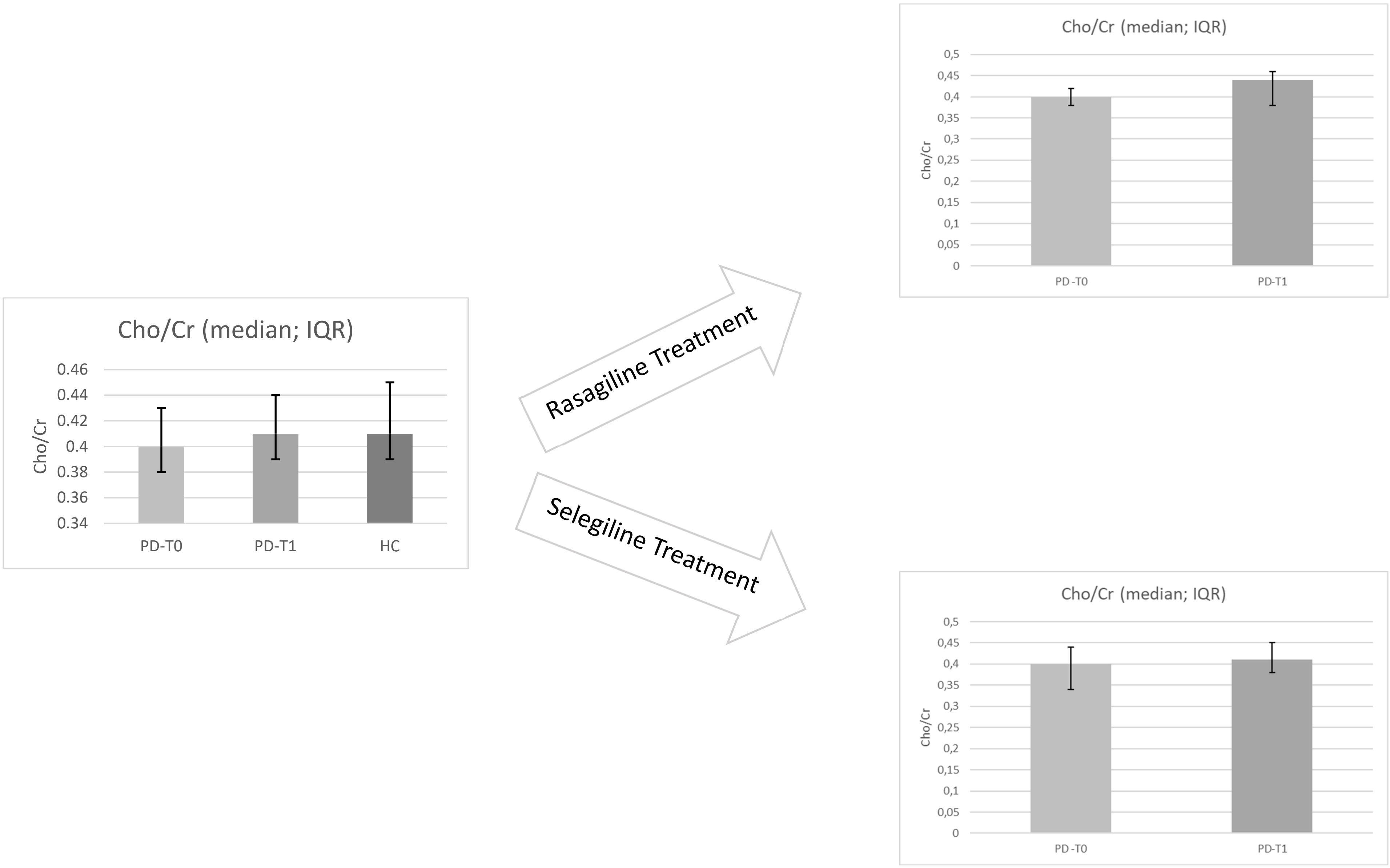
Cho/Cr (median; IQR)
0.46
0.44
0.42
0.4
0.38
0.36
0.34
Cho/Cr
PD-T0
PD-T1
HC
Rasagiline Treatment
Selegiline Treatment
Cho/Cr (median; IQR)
0,5
0,45
0,4
0,35
0,3
0,25
0,2
0,15
0,1
0,05
0
Cho/Cr
PD -T0
PD-T1
Cho/Cr (median; IQR)
0,5
0,45
0,4
0,35
0,3
0,25
0,2
0,15
0,1
0,05
0
Cho/Cr
PD -T0
PD-T1

Figure S2. Motor-cortex metabolite ratios in de novo PD under MAO-B inhibitors (Bonanno et al., J Clin Med 2022). Bar plots show NAA/Cr (top) and Cho/Cr (bottom) acquired with multi-voxel ^{1}H-MRS (3 T) over the bilateral motor cortex. Cohorts: PD-T0 = de novo, drug-naïve PD at baseline; PD-T1 = the same patients after 12 months of rasagiline or selegiline; HC = healthy controls (PD n=40; HC n=40). Bars = median; error bars = interquartile range (IQR). In the original study, NAA/Cr was reduced at PD-T0 vs HC and increased significantly at PD-T1, approaching HC values, whereas Cho/Cr showed no significant change.
Legend: PD= Parkinson's disease; HC= healthy controls; T0/T1, baseline/12-month follow-up.

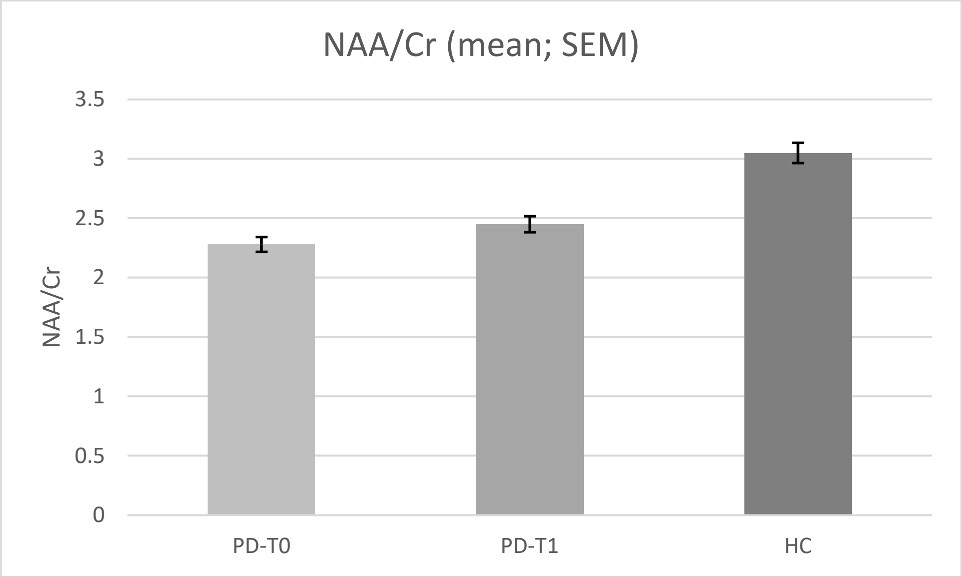
NAA/Cr (mean; SEM)
NAA/Cr
3.5
3
2.5
2
1.5
1
0.5
0
PD-T0
PD-T1
HC

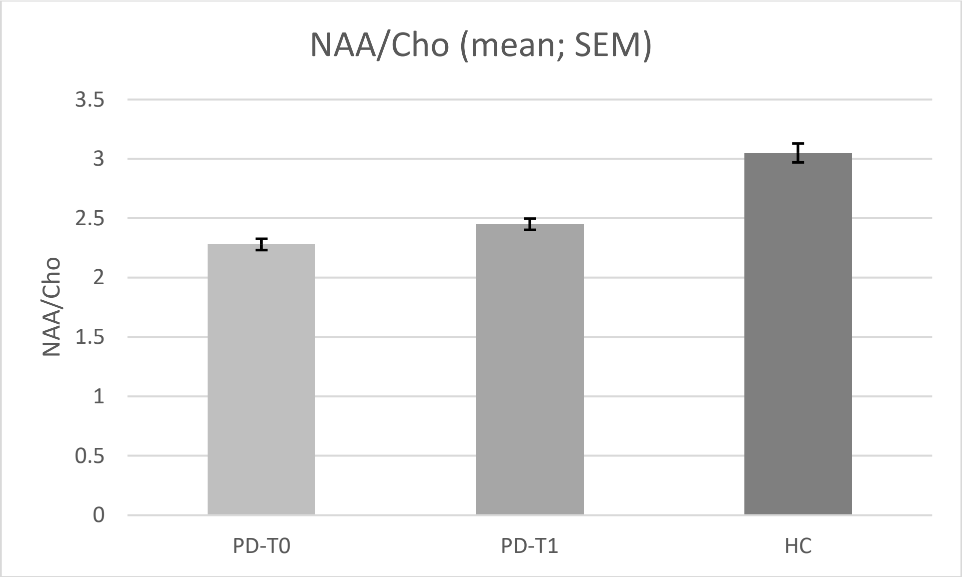
NAA/Cho (mean; SEM)
NAA/Cho
3.5
3
2.5
2
1.5
1
0.5
0
PD-T0
PD-T1
HC

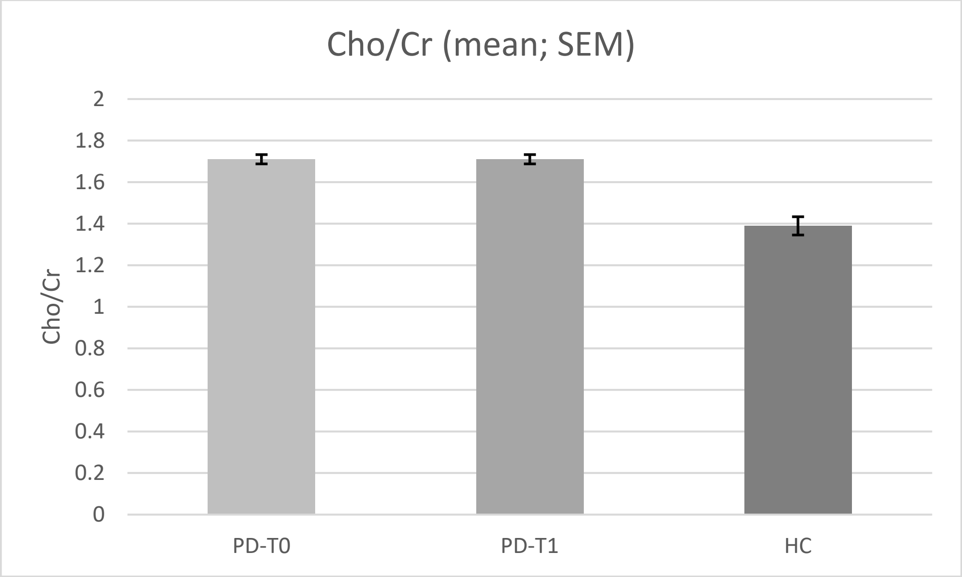
Cho/Cr (mean; SEM)
Cho/Cr
2
1.8
1.6
1.4
1.2
1
0.8
0.6
0.4
0.2
0
PD-T0
PD-T1
HC

Figure S3. Motor-cortex metabolite ratios before and after ropinirole (Ciurleo et al., Neurosci Lett 2015). Bar plots display NAA/Cr (top), NAA/Cho (middle), and Cho/Cr (bottom) measured with multi-voxel ^{1}H-MRS (1.5 T) over the mesial motor cortex. Groups: PD-T0 = de novo, drug-naïve PD at baseline; PD-T1 = the same patients after 10 months of ropinirole (up to 6 mg/day); HC = healthy controls (PD n=20; HC n=15). Bars = mean; error bars = SEM (from the study's summary statistics). In the original report, PD-T0 showed reduced NAA/Cr and NAA/Cho and increased Cho/Cr vs HC; after treatment, NAA/Cr and NAA/Cho significantly increased toward HC values, whereas Cho/Cr did not change significantly.
Legend: PD= Parkinson's disease; HC= healthy controls; T0/T1, baseline/10-month follow-up.

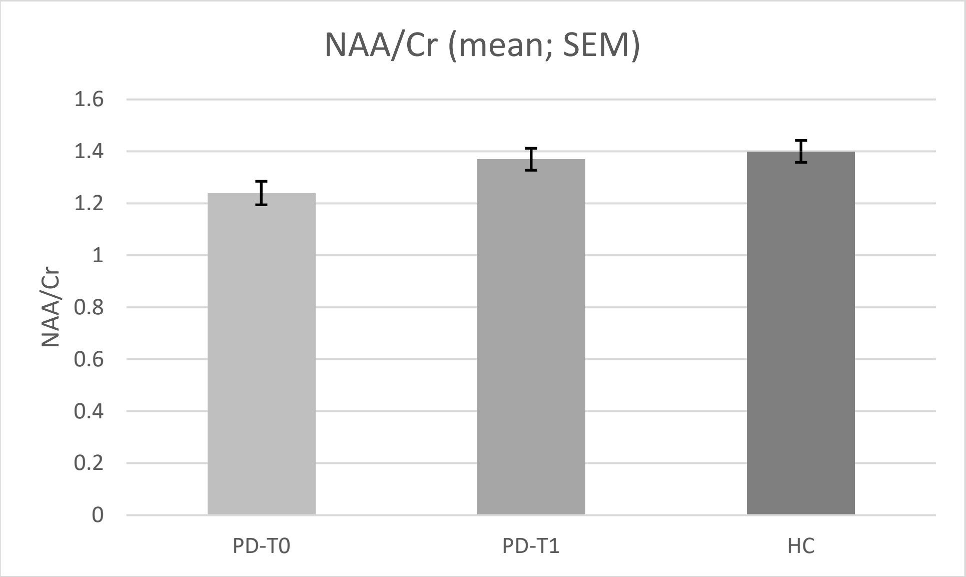
NAA/Cr (mean; SEM)
1.6
1.4
1.2
1
0.8
0.6
0.4
0.2
0
NAA/Cr
PD-T0
PD-T1
HC

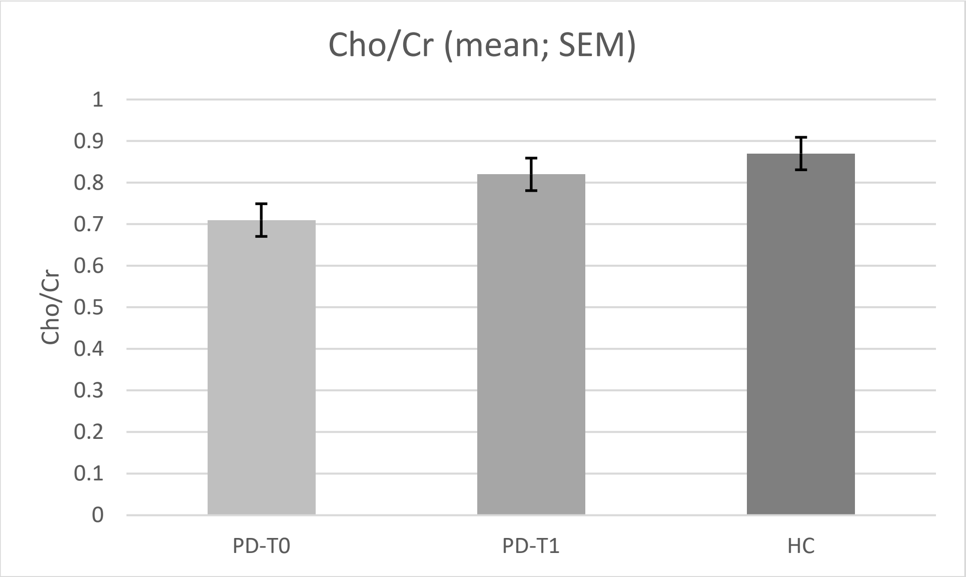
Cho/Cr (mean; SEM)
1
0.9
0.8
0.7
0.6
0.5
0.4
0.3
0.2
0.1
0
Cho/Cr
PD-T0
PD-T1
HC

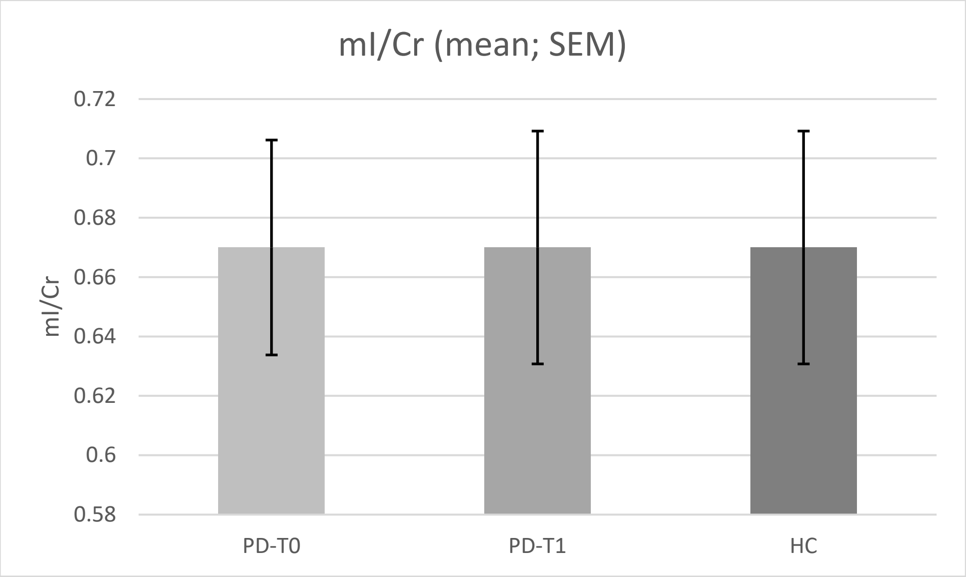
mI/Cr (mean; SEM)
0.72
0.7
0.68
0.66
0.64
0.62
0.6
0.58
mI/Cr
PD-T0
PD-T1
HC

Figure S4. Motor-cortex metabolite ratios before and after pergolide (Lucetti et al., Mov Disord 2007). Bar plots show NAA/Cr (top), Cho/Cr (middle), and mI/Cr (bottom) acquired with single-voxel ^{1}H-MRS (1.5 T) from the medial motor cortex. Groups: PD-T0 = de novo, drug-naïve PD at baseline; PD-T1 = the same patients after 6 months of pergolide (1 mg t.i.d.); HC = healthy controls (PD n=11; HC n=11). Bars = mean; error bars = SEM; data re-plotted from the published summary statistics. In the original report, PD-T0 showed reduced NAA/Cr and Cho/Cr vs HC; after treatment, Cho/Cr increased significantly, whereas NAA/Cr showed a non-significant upward trend and mI/Cr remained unchanged.
Legend: PD= Parkinson's disease; HC= healthy controls; T0/T1, baseline/6-month follow-up.
